# Morusin targeting GDF15 enhances ferroptosis and overcomes cisplatin resistance in NSCLC

**DOI:** 10.3389/fphar.2026.1803939

**Published:** 2026-06-19

**Authors:** Liang Zhang, Huan Liu, Si Jiang, Desheng Li, Xin Li, Maolei Xu, Jianwen Fei, Ling Zhou

**Affiliations:** 1 Department of Respiratory and Critical Care Medicine, Yantaishan Hospital Affiliated to Binzhou Medical University, Binzhou Medical University, Yantai, Shandong, China; 2 The Key Laboratory of Traditional Chinese Medicine Prescription Effect and Clinical Evaluation of State Administration of Traditional Chinese Medicine, School of Pharmacy, Binzhou Medical University, YanTai, Shandong, China

**Keywords:** cisplatin, ferroptosis, GDF15, morusin, NSCLC

## Abstract

**Background:**

The natural compound morusin (Mor) acts as a potent tumor suppressor in non-small cell lung cancer (NSCLC), but its potential to sensitize DDP and its direct targets are less understood. This work aims to investigate the DDP-sensitizing effects of Mor and the underlying mechanisms.

**Methods:**

Cell viability and drug synergy were assessed in NSCLC cells. Ferroptosis was evaluated by measuring lipid reactive oxygen species (ROS), iron accumulation, and the expression of ferroptosis markers. GDF15 was identified as a target via transcriptome sequencing. Its direct binding with Mor was confirmed by molecular docking. The ubiquitin-mediated degradation mechanism and its functional role in ferroptosis were validated using the proteasome inhibitor MG132, alongside GDF15 overexpression and knockdown models. Clinical relevance was assessed using TCGA database analysis.

**Results:**

Mor induces ferroptosis and significantly augments DDP sensitivity in both NSCLC and cisplatin-resistant A549 cells (A549/DDP). Mechanistically, Mor directly binds to GDF15 and promotes its ubiquitin-mediated degradation. Consequently, GDF15 overexpression reversed Mor-induced cytotoxicity and DDP sensitization. Furthermore, DDP exposure impairs intracellular GDF15 protein levels, and the Mor/DDP combination synergistically suppresses GDF15 in A549 cells. Notably, GDF15 expression is elevated in NSCLC cells compared to normal lung epithelial cells. However, A549/DDP cells exhibit diminished intracellular GDF15 protein relative to parental cells, while GDF15 transcription is upregulated. Knockdown of GDF15 augmented DDP sensitivity in resistant cells.

**Conclusion:**

Mor reverses DDP resistance by inducing GDF15 degradation and ferroptosis, suggesting that Mor-based combination therapy holds promise in treating NSCLC.

## 1 Introduction

Non-small cell lung cancer (NSCLC) is the most prevalent type of lung cancer, with its clinical management and biological mechanisms constituting the core of contemporary lung cancer studies (Hendriks et al., 2024). While treatment for advanced or metastatic NSCLC has evolved to include targeted therapies and immunotherapy as first-line treatments for specific patient populations (Cheng and Perez-Soler, 2018), platinum-based chemotherapy remains an indispensable backbone. It is frequently utilized either as a foundational combination therapy with immunomodulators or as a primary option for patients lacking actionable genetic mutations (Lisberg and Garon, 2019; Hendriks et al., 2023). However, the development of intrinsic or acquired resistance to chemotherapeutic agents, such as cisplatin (DDP), continues to be a primary cause of treatment failure and a major clinical challenge.

Ferroptosis is a mode of cell death characterized by elevated lipid peroxidation, intracellular iron accumulation, and the generation of reactive oxygen species (ROS). Increasing evidence suggests that ferroptosis inducers can significantly enhance the sensitivity of tumor cells to chemotherapy (Zhang et al., 2023; Zhang et al., 2023), highlighting the importance of ferroptosis in managing drug-resistant tumors. Emerging data indicate that DDP induces ferroptosis and lipid peroxide accumulation (Kim et al., 2022; Kim et al., 2022); thus, promoting ferroptotic cell death serves as a promising strategy to overcome DDP resistance.

Growth and differentiation factor 15 (GDF15), a stress-responsive cytokine belonging to the transforming growth factor-β (TGF-β) superfamily, exerts pleiotropic physiological and pathological effects. In cancer, GDF15 functions as a critical survival factor that drives tumorigenesis and chemoresistance by shifting cellular redox status and bolstering antioxidant defenses to suppress ferroptosis (Siddiqui et al., 2022; Dai et al., 2024; Huang Fu et al., 2024; Guo et al., 2025). Conversely, targeted downregulation of GDF15 overcomes this resistance, inhibiting tumor growth and metastasis while sensitizing cancer cells to ferroptosis-inducing agents such as erastin (Chen et al., 2020; Xiang et al., 2025). Mechanistically, upon induction by chemotherapeutic stress, GDF15 is robustly secreted into the extracellular milieu to act as an autocrine or paracrine signaling molecule (Worth et al., 2020). By binding to cognate surface receptors, such as TGFBR2, secretory GDF15 triggers downstream kinase cascades to stabilize antioxidant regulators (Okamoto et al., 2020). This receptor-mediated signaling efficiently scavenges intracellular ROS and mitigates lipid peroxidation (Feng et al., 2025), which, alongside the reprogramming of fatty acid metabolism, ultimately confers resistance to ferroptosis and therapy-induced cell death (Wang et al., 2021).

Morusin (Mor), a natural flavonoid compound, has recently garnered significant attention for its anti-tumor activity (Hafeez et al., 2023; Li et al., 2024). Studies have shown that Mor exerts its anti-NSCLC effects by inhibiting proliferation and metastasis, activating autophagy, and inducing apoptosis. Mechanistic studies revealed that Mor’s anti-NSCLC effects are partly mediated through the activation of AMP-activated protein kinase, inhibition of the EGFR/STAT3 signaling pathway and PI3K/Akt signaling (Park et al., 2018; Park and Park, 2020; Wang et al., 2020). However, it remains unclear whether Mor treatment can induce ferroptosis, and the direct targets of Mor in its anti-NSCLC effects have not yet been fully elucidated.

This work aimed to determine the DDP-sensitizing effects of Mor and its underlying mechanisms in NSCLC. We showed here that Mor significantly augmented DDP sensitivity in both parental and resistant NSCLC cells by inducing ferroptosis. Mechanistically, Mor directly bound to GDF15 and promoted its ubiquitin-mediated degradation, thereby abrogating GDF15-dependent chemoresistance. Our findings present Mor as a potent ferroptosis inducer and a promising combination therapy candidate for overcoming DDP resistance in NSCLC.

## 2 Materials and methods

### 2.1 Reagents and antibodies

Morusin (purity ≥98.0%, ST15820120) was obtained from Nature Standard Company (Shanghai, China). Cisplatin (C295225) were purchased from Shanghai Aladdin Biochemical Technology Co., Ltd. Chloroquine (HY-17589A), Ferrostatin-1 (HY-100579), z-VAD-fmk (HY-16658B), and MG-132 (HY-13259) were purchased from MCE (Shanghai, China). Primary antibodies targeting P62/SQSTM1 (18420-1-AP), LC3 (14600-1-AP), FTH1 (11682-1-AP), GPX4 (67763-1-Ig), GDF15 (27455-1-AP), and β-actin (20536-1-AP) were acquired from Proteintech (Wuhan Sanying Biotechnology, China).

### 2.2 Cell lines and cultures

The NSCLC cell lines A549 and H1975, along with the normal human non-epithelial cell line Beas-2B, were sourced from the Cell Bank of the Chinese Academy of Sciences. The cisplatin-resistant A549 cell line (A549/DDP) were purchased from Procell Life Science & Technology Co., Ltd. (Wuhan, China; Catalog No.CL-0519). The NSCLC cell lines were cultured in RPMI 1640 medium supplemented with 10% fetal bovine serum (FBS) and 1% penicillin/streptomycin, whereas Beas-2B cells were maintained in DMEM medium. To maintain the drug-resistant phenotype, A549/DDP cells were continuously cultured in the presence of 2 μg/mL cisplatin. All cell lines were incubated in a humidified environment at 37 °C with 5% CO2.

### 2.3 Cell proliferation analysis and synergy calculation

The anti-proliferative effects of Mor and DDP were evaluated using the MTT assay. Briefly, NSCLC cells were seeded into 96-well plates at a density of 5 × 10^3^ cells per well. Following this, the cells were treated with a range of concentrations of Mor, DDP alone, or their combinations for the specified durations. After the treatment periods, MTT solution (0.5 mg/mL) was added to each well and incubated for 4 h at 37 °C. The formazan crystals formed were subsequently dissolved in dimethyl sulfoxide, and the absorbance was measured at 570 nm using a microplate reader. Cell viability was expressed as a percentage relative to the untreated control group. The half-maximal inhibitory concentration (IC_50_) values of Mor and DDP for each cell line were calculated using GraphPad Prism software (Version 10.0, GraphPad Software, United States).

The potential synergistic interaction between Mor and DDP was quantitatively analyzed using CalcuSyn software (version 2.0, Biosoft, Cambridge, United Kingdom), which is based on the Chou-Talalay method. According to the classic definition, a combination index (CI) value of <1, = 1, and >1 indicates synergy, an additive effect, and antagonism, respectively.

### 2.4 Western blotting analysis

Western blotting was conducted following standard protocols (Yang et al., 2023). After treatment with Mor, DDP alone, or their combination, the cells were harvested. Total protein was extracted using RIPA lysis buffer, and the protein concentration was determined using a BCA protein assay kit. Equal amounts of protein (20–50 µg) were separated by SDS-PAGE on 10% or 12% gels and subsequently transferred to PVDF membranes. Following a blocking step with 5% non-fat milk, the membranes were incubated overnight at 4 °C with specific primary antibodies. After washing, the membranes were incubated with HRP-conjugated secondary antibodies for 2 h at room temperature. The bands were visualized using enhanced chemiluminescence (Tanon, Shanghai, China) with the Tanon 5200 chemiluminescence imaging system (Tanon). Protein bands were quantified with ImageJ software (version 1.53, National Institutes of Health, Bethesda, MD, United States).

### 2.5 Immunofluorescence staining

NSCLC cells were seeded onto glass coverslips placed in 24-well plates and allowed to adhere overnight as mentioned earlier (Xu et al., 2024). Following treatment with Mor, the cells were fixed using 4% paraformaldehyde for 5 min at room temperature, permeabilized with 0.1% Triton X-100 for 10 min, and subsequently blocked with 5% goat serum for 1 h. The cells were then incubated overnight at 4 °C with a primary antibody against LC3, followed by a 2 h incubation at room temperature with a fluorophore-conjugated secondary antibody. Cell nuclei were counterstained with DAPI for 5 min. After sealing, images were captured using a laser scanning confocal microscope. The formation of autophagosomes was evaluated by observing the characteristic punctate fluorescence pattern of LC3-II (green dots).

### 2.6 Apoptosis

Apoptosis was assessed using an Annexin V-FITC/PI apoptosis detection kit (Absin Bioscience Inc, Shanghai, China). In brief, non-small cell lung cancer (NSCLC) cells were treated with Mor for 24 h. Following treatment, the cells were collected, washed, and resuspended in 100 µL of 1× binding buffer. The cell suspension was then incubated with Annexin V-FITC and propidium iodide (PI) for 10–15 min. After incubation, 400 µL of 1× binding buffer was added. Analysis was conducted using a FACS Canto flow cytometer (BD Biosciences), and data were analyzed with FlowJo software to differentiate between viable (Annexin V^−^/PI^−^), early apoptotic (Annexin V^+^/PI^−^), late apoptotic (Annexin V^+^/PI^+^), and necrotic (Annexin V^−^/PI^+^) cell populations.

### 2.7 ROS and lipid peroxidation assay

Intracellular ROS and lipid peroxidation levels were quantified using fluorescent probes and flow cytometry according to previous research (Zhou et al., 2021). Following a 24 h treatment with Mor, NSCLC cells were harvested, washed, and incubated with the respective probes in serum-free medium at 37 °C for 30 min. Specifically, total ROS was measured using 10 μM DCFH-DA (Beyotime Biotechnology, Shanghai, China), while lipid ROS was evaluated using 2 μM BODIPY™ 581/591 C11 (Thermo Scientific, Waltham, MA, United States). After incubation, the cells were washed, resuspended, and analyzed immediately via flow cytometry. Data processing and analysis were conducted using FlowJo software, where the mean fluorescence intensity (MFI) was quantified.

### 2.8 Detection of Intracellular Fe^2+^

Intracellular iron levels were assessed using FerroOrange (Dojindo, Kumamoto, Japan). Cells were seeded in 6-well plates and subjected to the specified treatment. After 24 h, FerroOrange (1 μM) diluted in serum-free medium was added to the cells, followed by a 30-min incubation at 37 °C. Finally, fluorescence images were obtained using a confocal microscope.

### 2.9 RNA-Seq transcriptome analysis

Total RNA was extracted from A549 cells treated with or without Mor using TRIzol® Reagent, following the manufacturer’s instructions. The integrity and concentration of the RNA were assessed. Subsequently, RNA samples were sent for library preparation and sequencing on the Illumina NovaSeq 6000 platform by Shanghai Majorbio Bio-Pharm Technology. These analyses were conducted using the online Majorbio I-Sanger Cloud Platform (https://cloud.majorbio.com/). The dataset is publicly accessible via the following DOI/link: https://doi.org/10.5281/zenodo.20148802.

### 2.10 Quantitative real-time PCR analysis

Total RNA was extracted from treated cells using TRIzol® Reagent and reverse-transcribed to cDNA with the Evo M-MLV RT Premix kit (Cat. No. AG11706, Accurate Biology, China). RT-qPCR was conducted using the SYBR Green Premix Pro Taq HS qPCR kit (Cat. No. AG11701, Accurate Biotechnology, Hunan, China). The relative expression of target genes, normalized to β-actin, was calculated using the 2 ^–ΔΔ Ct^ method. The following primers were employed to amplify the target genes: GDF15 (forward), 5′-GCA​AGA​ACT​CAG​GAC​GGT​GA-3′; GDF15 (reverse), 5′-TGG​AGT​CTT​CGG​AGT​GCA​AC-3′.

### 2.11 Transfection in NSCLC cells

For GDF15 knockdown, A549/DDP cells were transfected with specific siRNA using Lipofectamine ® 3000 transfection reagent (Invitrogen, United States). Negative siRNA was used as a normal control. For GDF15 overexpression, A549 cells were transfected with GDF15 plasmids according to the manufacturer’s protocol. Empty vectors (pcDNA3.1) were transfected as control. Briefly, the culture medium was changed to Opti-MEM prior to transfection, and the mixture of Lipofectamine® 3000 and each siRNA or plasmids pool were added to the cells. Media containing 10% FBS was added to the cells after 6 h incubation at 37 °C. The GDF15 siRNA oligos used are as follows: sense, 5′-CUC​GAG​UCC​AAC​GAG​UGU​CTT-3'; antisense, 5′-GAC​ACU​CGU​UGG​ACU​CGA​GTT-3'.

### 2.12 Molecular docking

Molecular docking was conducted using AutoDock 4.2 to simulate the interaction between Mor and GDF15 (PDB ID: 5VZ4). The protein structure was prepared by removing water molecules and adding hydrogen atoms. The three-dimensional structure of Mor underwent energy minimization. Docking simulations were performed using the Lamarckian genetic algorithm, and the resulting binding poses were ranked according to their calculated binding affinities (kcal/mol). The optimal binding mode was further analyzed and visualized for intermolecular interactions using Discovery Studio Visualizer.

### 2.13 Correlation between GDF15 and ferroptosis suppressor gene set in NSCLC samples

We downloaded STAR-counts data along with corresponding clinical information for NSCLC tumors from the TCGA database (https://portal.gdc.cancer.gov). Subsequently, we extracted the data in Transcripts Per Million (TPM) format and performed normalization using the log2 (TPM +1) transformation. After filtering for samples that contained both RNA sequencing data and clinical information, we ultimately selected 261 samples for further analysis. The ferroptosis-related genes were derived from a systematic analysis of the abnormalities and functions of ferroptosis in cancer. Statistical analyses were conducted using R software, version 4.0.3, with results deemed statistically significant when the p-value was less than 0.05.

### 2.14 Statistical analysis

All data are presented as the mean ± SEM from at least three independent experiments. Statistical comparisons between two groups were performed using Student’s t-test. For comparisons involving three or more groups, one-way ANOVA followed by Tukey’s post hoc test was employed. In the bioinformatics analysis, differentially expressed genes between the two conditions were identified using the Wilcoxon rank-sum test. A p-value of less than 0.05 was deemed statistically significant. All statistical analyses were conducted using GraphPad Prism software.

## 3 Results

### 3.1 Mor induces ferroptosis in NSCLC cells

**FIGURE 1:**
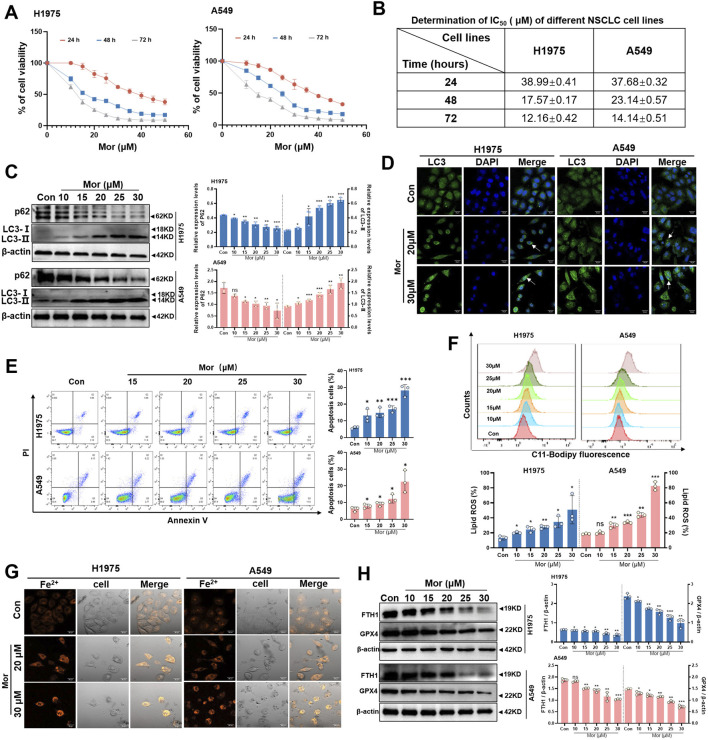
Mor induces ferroptosis in NSCLC cells. **(A)** Viability of NSCLC cells treated with the indicated concentrations of Mor for 24, 48, and 72 h **(B)** IC_50_ values of Mor in NSCLC cells at 24, 48, and 72 h. **(C)** Western blot analysis of autophagy markers LC3-II and p62 in NSCLC cells after Mor treatment for 24 h. **(D)** Representative immunofluorescence images showing LC3 puncta (green) formation in cells treated with Mor for 24 h (Scale bar, 20 μm). **(E)** Flow cytometry analysis of apoptosis using Annexin V-FITC/PI staining in cells treated with Mor for 24 h. **(F)** Lipid ROS levels in cells treated with Mor for 24 h. The shift in fluorescence indicates increased lipid peroxidation. **(G)** Intracellular Fe^2+^ levels in cells treated with Mor for 24 h. Red fluorescence intensity correlates with Fe^2+^ accumulation. **(H)** Western blot analysis of GPX4 and FTH1 in NSCLC cells after Mor treatment for 24 h. For **(C,H)**, β-actin served as a loading control. Values represent mean ± SD. ns, not significant, *p < 0.05, **p < 0.01, ***p < 0.001 vs. Con group.

Initial assays revealed that Mor inhibits the viability of NSCLC cells A549 and H1975 in a concentration-dependent manner (Figure 1A), with the corresponding IC_50_ values determined at three different time points (24 h, 48 h, and 72 h) (Figure 1B). Consistent with previous reports, the ability of Mor to induce autophagy was confirmed by alterations in the expression of autophagy-related proteins and autophagic flux (Figures 1C,D), alongside the induction of apoptosis verified via Annexin V/PI double staining (Figure 1E). To determine if ferroptosis contributes to Mor-mediated cytotoxicity, we next evaluated the biochemical markers of ferroptosis. Notably, Mor exposure significantly promoted increased lipid ROS generation (Figure 1F) and iron accumulation (Figure 1G) in A549 and H1975 cells. In close agreement with these observations, the protein expressions of GPX4 and FTH1 were markedly downregulated in cells treated with Mor compared to control cells (Figure 1H). Collectively, these results suggest that Mor-induced ferroptosis may represent an additional therapeutic pathway for combating NSCLC.

### 3.2 Mor enhances the chemosensitivity of NSCLC cells to DDP

**FIGURE 2:**
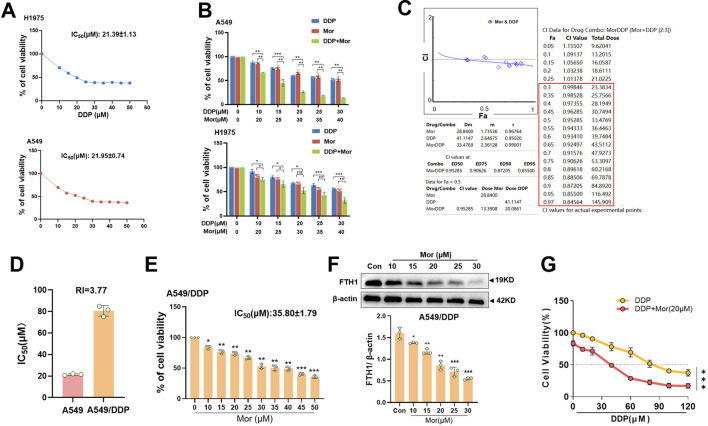
Mor sensitizes NSCLC cells to DDP. **(A)** IC_50_ values of DDP in NSCLC cells at 24 h. **(B)** Viability of NSCLC cells treated with Mor, DDP, or their combination for 24 h. **(C)** Combination index (CI) of Mor and DDP calculated using the Chou-Talalay method. CI < 1 indicates synergy. **(D)** Resistance index (RI) of A549/DDP cells relative to parental A549 cells. **(E)** Viability and corresponding IC_50_ value of A549/DDP cells treated with the indicated concentrations of Mor for 24 h. **(F)** Western blot analysis of FTH1 in A549/DDP cells following Mor treatment for 24 h. β-actin served as a loading control. **(G)** IC_50_ values of DDP in A549/DDP cells with or without Mor co-treatment for 24 h. Values represent mean ± SD. For **(B)**, ns, no significant, *p < 0.05, **p < 0.01, ***p < 0.001. For **(E)** and **(F)**, *p < 0.05, **p < 0.01, ***p < 0.001 vs. Con group. For **(G)**, ***p < 0.001 vs. DDP alone group.

We subsequently investigated whether Mor could enhance the sensitivity of NSCLC to DDP. Initially, we assessed the effects of DDP on two human NSCLC cell lines, A549 and H1975, with the IC_50_ values presented in Figure 2A. The drug combination ratio was established based on the IC_50_ values of each single agent in the respective cell lines. Notably, combined treatment significantly reduced cell viability compared to monotherapy with either Mor or DDP alone in both A549 and H1975 cells (Figure 2B). The CI values at varying fractional effect (Fa) levels were calculated using the median-effect points of the single drugs or the drug combinations at fixed ratios. The combination of Mor and DDP exhibited a synergistic effect in A549 cells, with CI values consistently below 1 across Fa values ranging from 0.3 to 0.97 (Figure 2C). The IC_50_ values for A549 and A549/DDP cells were determined to be 21.95 ± 0.74 μM and 80.66 ± 4.76 μM, respectively, resulting in a resistance index of 3.77 (Figure 2D). Mor alone also inhibited the proliferation of A549/DDP cells, with an IC_50_ value of 35.80 ± 1.97 μM (Figure 2E). In addition, we observed that Mor effectively inhibited the expression of FTH1 in A549/DDP cells (Figure 2F). Specifically, treatment with 20 μM Mor markedly enhanced A549/DDP cells sensitivity to DDP, as evidenced by a reduced IC_50_ of DDP in A549/DDP cells (Figure 2G).

### 3.3 Mor sensitizes NSCLC cells to cisplatin via the induction of ferroptosis

**FIGURE 3:**
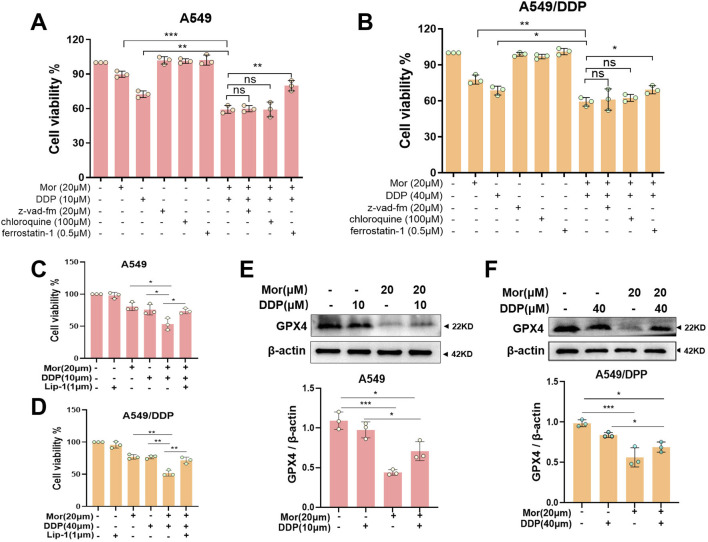
Mor sensitizes NSCLC cells to cisplatin via the induction of ferroptosis. **(A,B)** Viability of A549 **(A)** and A549/DDP **(B)** cells co-treated with various cell death inhibitors, Mor, and DDP for 24 h **(C,D)** Viability of A549 **(C)** and A549/DDP **(D)** cells treated with Mor and DDP in the presence or absence of the ferroptosis inhibitor Liproxstatin-1 (Lip-1) for 24 h **(E,F)** Western blot analysis of GPX4 in A549 **(E)** and A549/DDP **(F)** cells following treatment with Mor, DDP, or their combination for 24 h β-actin served as a loading control. Values represent mean ± SD. ns, not significant, *p < 0.05, **p < 0.01, ***p < 0.001.

To investigate the underlying mechanisms by which Mor augments the chemosensitivity of NSCLC cells to DDP, we employed specific inhibitors targeting distinct cell death pathways, including z-VAD-FMK (apoptosis), chloroquine (autophagy), and ferrostatin-1 (ferroptosis). Notably, while apoptosis and autophagy inhibitors failed to reverse the enhanced cytotoxicity, the ferroptosis inhibitor ferrostatin-1 effectively prevented the Mor-mediated sensitization to DDP (Figure 3A). Similar to what was observed in parental cells, this ferroptosis-dependent sensitization was replicated in A549/DDP cells (Figure 3B). We performed additional rescue assays utilizing another ferroptosis inhibitor, liproxstatin-1 (Lip-1). Corroborating these results, Lip-1 treatment significantly mitigated Mor-induced cytotoxicity (Figures 3C,D), suggesting that Mor augments DDP sensitivity primarily through the induction of ferroptosis. Furthermore, we evaluated GPX4 expression following treatment with DDP, Mor, or their combination. Consistently across both cell lines, GPX4 protein was significantly suppressed by Mor alone and the combined treatment (Figures 3E,F). Furthermore, compared with DDP monotherapy, the Mor and DDP combination yielded a profound reduction in GPX4 levels in both A549 and A549/DDP cells (Figures 3E,F).

### 3.4 Mor inhibits GDF15 in NSCLC A549 cells

**FIGURE 4:**
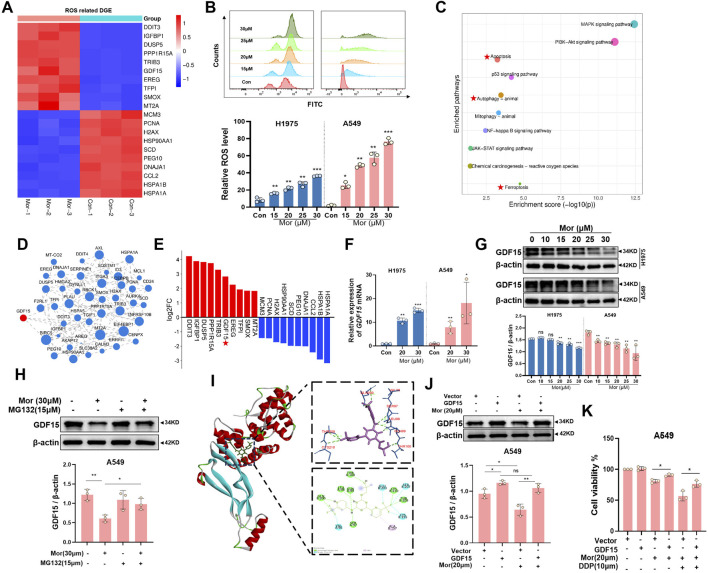
Mor induces oxidative stress and inhibits GDF15 in A549 cells. **(A)** Heatmap of differentially expressed ROS-related genes in A549 cells following Mor treatment for 24 h. **(B)** Intracellular ROS levels in NSCLC cells treated with Mor for 24 h **(C)** KEGG pathway enrichment analysis of the differentially expressed ROS-related genes. **(D)** Interaction network of the differentially expressed ROS-related genes, highlighting the core hub genes. **(E)** The top 10 upregulated and downregulated genes in A549 cells treated with Mor for 24 h. **(F)** Relative mRNA levels of GDF15 in NSCLC cells treated with Mor for 24 h. **(G)** Western blot analysis of GDF15 in NSCLC cells following Mor treatment for 24 h. **(H)** Western blot analysis of GDF15 in NSCLC cells pretreated with the proteasome inhibitor MG132 prior to Mor treatment for 24 h. **(I)** Predicted binding mode and key hydrogen-bond interactions between Mor and GDF15 obtained via molecular docking simulation. **(J)** Western blot analysis confirming the efficiency of GDF15 overexpression and its effect on Mor-induced GDF15 reduction in A549 cells treated for 24 h. **(K)** Viability of A549 cells transfected with the GDF15 overexpression plasmid or empty vector following treatment with Mor alone or in combination with DDP for 24 h. For **(G)**, **(H)**, and **(J)**, β-actin served as a loading control. Values represent mean ± SD. ns, not significant, *p < 0.05, **p < 0.01, ***p < 0.001. For **(B)**, **(F)**, and **(G)**, statistical significance was determined compared to the Con group.

There is increasing evidence that the generation of ROS is closely associated with autophagy, apoptosis, and ferroptosis (Wang et al., 2023a; Wang et al., 2023b). Transcriptome analysis has revealed significant alterations in the expression of genes related to ROS (Figure 4A). Consistent with these transcriptomic profiles, subsequent fluorometric assessments confirmed that Mor exposure markedly augmented intracellular ROS accumulation (Figure 4B). Additionally, we conducted KEGG pathway enrichment analysis on the differentially expressed genes associated with ROS and found that these genes were also enriched in the pathways of ferroptosis, apoptosis, and autophagy (Figure 4C). Construction of a molecular interaction network based on these genes identified GDF15 as a core hub (Figure 4D). Furthermore, among the top 20 differentially expressed ROS-related genes, GDF15 exhibited one of the highest fold changes (Figure 4E). Previous reports suggest that the inhibition of GDF15 contributes to erastin-stimulated ferroptosis (Chen et al., 2020). Accordingly, we hypothesize that GDF15 may play a crucial role in the anti-cancer effects of Mor on NSCLC cells. To validate these findings, we performed an RT-PCR analysis and observed that, compared to the control group, the GDF15 mRNA level was significantly upregulated 24 h after Mor treatment (Figure 4F), which aligns with the results from transcriptome sequencing. However, Mor decreased GDF15 protein levels (Figure 4G). This discrepancy indicates that Mor modulates GDF15 via post-translational regulation. Indeed, pretreatment with the proteasome inhibitor MG132 reversed the Mor-mediated reduction of GDF15 protein (Figure 4H), suggesting that Mor induces GDF15 degradation via the ubiquitin-proteasome pathway. Molecular docking analysis further supported a direct interaction between Mor and GDF15. The predicted binding mode revealed a favorable docking score (−8.1 kcal/mol), which involved specific hydrogen-bonds with key GDF15 residues Gln200, Leu98, Gln99, and Thr100 (Figure 4I). We further investigated the role of GDF15 in Mor-induced ferroptosis by ectopically overexpressing GDF15 in A549 cells. GDF15 overexpression counteracted the Mor-mediated degradation of GDF15 (Figure 4J). Consistently, ectopic GDF15 rescued the cell viability impaired by Mor monotherapy or the Mor/DDP combination (Figure 4K). These results indicate that GDF15 downregulation partially mediates Mor-induced ferroptosis in NSCLC cells.

### 3.5 Cisplatin reduces intracellular GDF15 protein levels in NSCLC cells

**FIGURE 5:**
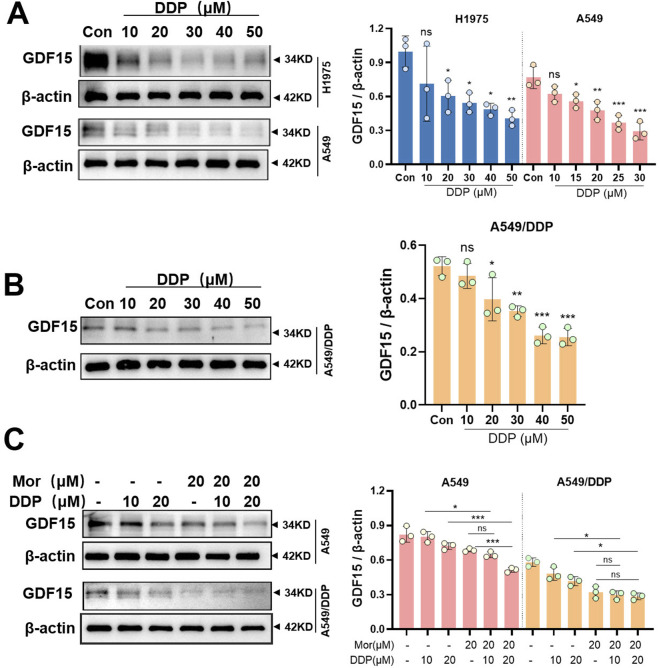
Cisplatin reduces intracellular GDF15 protein levels in NSCLC cells. **(A,B)** Western blot analysis of GDF15 in A549, H1975 **(A)** and A549/DDP **(B)** cells following DDP treatment for 24 h. **(C)** Western blot analysis of GDF15 in A549 and A549/DDP cells treated with Mor, DDP, or their combination for 24 h. For **(A–C)**, β-actin served as a loading control. Values represent mean ± SD. ns, not significant, *p < 0.05, **p < 0.01, ***p < 0.001. For **(A)** and **(B)**, statistical significance was determined compared to the Con group.

A recent study revealed that systemic DDP administration can induce GDF15 expression *in vivo* (Borner et al., 2020). However, how DDP directly modulates the intracellular GDF15 protein within NSCLC cells remains largely unexplored. Our results demonstrated a significant decrease in GDF15 levels following DDP treatment in NSCLC cells (Figure 5A). Similar findings were also noted in A549/DDP cells (Figure 5B). We subsequently assessed the effects of a combination therapy involving Mor and DDP on intracellular GDF15 protein levels. Treatment with Mor and DDP, either alone or in combination, downregulated GDF15 protein levels in both A549 and A549/DDP cells (Figure 5C). Notably, the combination therapy group exhibited a significantly greater reduction in GDF15 levels compared to the monotherapy groups in A549 cells. Conversely, in the A549/DDP cells, Mor and DDP individually caused a marked decrease of GDF15. Consequently, no further significant reduction was observed in the combination group (Figure 5C).

### 3.6 Inhibition of GDF15 enhances the sensitivity of NSCLC cells to DDP

**FIGURE 6:**
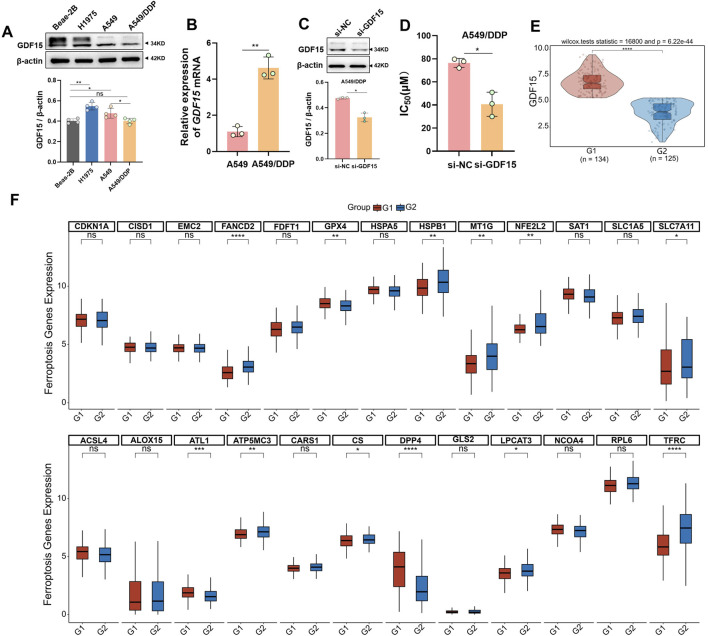
Inhibition of GDF15 enhances the sensitivity of NSCLC to DDP. **(A)** Western blot analysis of GDF15 in Beas-2B, H1975, A549, and A549/DDP cells. **(B)** Relative *GDF15* mRNA levels in A549 and A549/DDP cells determined by RT-qPCR. **(C)** Validation of GDF15 knockdown efficiency in A549/DDP cells transfected with GDF15 siRNA. **(D)** IC_50_ values of DDP in A549/DDP cells transfected with negative control (si-NC) or GDF15 siRNA following DDP treatment for 24 h. **(E)** Stratification of NSCLC patients from the TCGA database into *GDF15*-high and *GDF15*-low expression cohorts based on the median *GDF15* transcriptional level. **(F)** Correlation analysis between *GDF15* expression and key ferroptosis inhibitor genes in the TCGA cohort. For **(A,C)**, β-actin served as a loading control. Values represent mean ± SD. ns, not significant, *p < 0.05, **p < 0.01, ***p < 0.001, ****p < 0.0001.

Previous studies have demonstrated that GDF15 is aberrantly expressed in various tumor tissues (Mahon et al., 2024; Guan et al., 2025). Initially, we assessed the expression of GDF15 in human lung epithelial BEAS-2B cells and NSCLC cells. GDF15 expression was elevated in NSCLC cells compared with normal lung cells (Figure 6A). In contrast, intracellular GDF15 protein levels were significantly decreased in A549/DDP cells compared with parental A549 cells (Figure 6A). Previous studies have established that DDP exposure triggers its secretion into the extracellular space, where it activates membrane receptors to drive chemoresistance (Wang et al., 2023c). Therefore, we further examined *GDF15* mRNA expression in A549 and A549/DDP cells. A significant increase in *GDF15* mRNA was observed in A549/DDP cells (Figure 6B), suggesting that the low intracellular GDF15 protein abundance likely reflects an enhanced secretory characteristic of the resistant phenotype. To investigate the potential implication of GDF15 in chemoresistance, we silenced GDF15 in A549/DDP cells using siRNAs. GDF15 knockdown significantly reduced the IC_50_ value of DDP in A549/DDP cells (Figures 6C,D). In addition, we obtained *GDF15* gene expression data and corresponding clinical information for NSCLC from the TCGA database. Using the median transcriptional level of *GDF15* in NSCLC tissues as the cutoff, patients were stratified into high and low *GDF15* expression groups (Figure 6E). High *GDF15* expression positively correlated with elevated levels of GPX4, a master suppressor of ferroptosis, and DPP4. Conversely, low *GDF15* expression was associated with the upregulation of other iron metabolism and antioxidant genes (such as *FANCD2*, *HSPB1*, and *SLC7A11*), possibly reflecting a compensatory network (Figure 6F). Collectively, these data indicate that inhibition of GDF15 effectively enhances the sensitivity of NSCLC to DDP.

## 4 Discussion

In this work, we investigated the DDP-sensitizing effects of the natural compound Mor and its underlying mechanisms in NSCLC. The major findings of this work include: (1) Mor induces ferroptosis and significantly augments DDP sensitivity in both NSCLC and DDP-resistant NSCLC cells. (2) Mor directly binds to GDF15 and promotes its ubiquitin-mediated degradation, which is essential for Mor-induced cytotoxicity and DDP sensitization. (3) GDF15 is aberrantly expressed in NSCLC and serves as a driver of chemoresistance, while its targeted suppression effectively restores DDP sensitivity. Our data point to a potential therapeutic strategy of utilizing Mor-based combination regimens or targeting GDF15 to overcome DDP resistance in the treatment of NSCLC.

Currently, DDP resistance poses a major clinical challenge to the successful treatment of NSCLC. Emerging evidence highlights that targeting ferroptosis—a distinct form of cell death driven by lipid peroxidation—represents a promising strategy to overcome chemotherapeutic resistance (Lei et al., 2022; Zhang et al., 2022; Wang et al., 2023e). Furthermore, the pharmacological induction of ferroptosis by bioactive natural compounds, alongside the intricate post-translational modifications governing these processes, have increasingly become a focal point in reversing chemoresistance (Wang et al., 2023d; Wang et al., 2024). For instance, ferroptosis inducers RSL3 have been shown to enhance the anticancer effect of DDP (Li et al., 2021). While previous studies have established that Mor exerts anti-tumor effects by inducing apoptosis and autophagy, its potential to overcome chemoresistance remained undefined. In this work, we identified Mor as a ferroptosis inducer capable of overcoming DDP chemoresistance.

To elucidate the underlying mechanisms and identify specific drug targets, we performed a transcriptome analysis focusing on ROS-associated pathways, given that Mor upregulates intracellular ROS levels in NSCLC cells. Among the differentially expressed genes, GDF15 emerged as a central node. Previous studies have demonstrated that GDF15 is elevated in certain tumors and correlates with poor clinical outcomes (Zhu et al., 2024; Guan et al., 2025). Given the role of GDF15 in maintaining redox homeostasis, its downregulation accounts for the ROS accumulation and subsequent lipid peroxidation observed in Mor-treated NSCLC cells. Furthermore, GDF15 downregulation has been reported to promote ferroptosis by inhibiting SLC7A11 expression (Chen et al., 2020; Huang Fu et al., 2024). Consistently, our findings demonstrate that Mor promotes the post-translational degradation of GDF15 to drive ferroptosis, thereby reversing DDP resistance. As a consequence, the co-treatment of Mor and DDP significantly increased the sensitivity of both parental and DDP-resistant NSCLC cells. Consistently, ectopic GDF15 rescued the cell viability impaired by Mor monotherapy or the Mor/DDP combination. Taken together, these results indicate that Mor triggers ferroptosis and resensitizes NSCLC cells to DDP in a GDF15-dependent manner.

An increasing body of research indicates that DDP can induce the expression and secretion of GDF15, which contributes to the development of chemoresistance and immunosuppressive alterations in the tumor microenvironment (Du et al., 2023; Akdogan et al., 2024). Previous *in vivo* studies have established that systemic DDP administration profoundly increases circulating GDF15 levels as a systemic stress response to mediate chemotherapy-induced anorexia and emesis (Breen et al., 2020). However, our *in vitro* data appear different from this view. We observed that while GDF15 mRNA is aberrantly overexpressed in parental NSCLC cell lines, its intracellular protein levels are significantly downregulated in DDP-resistant cells. Furthermore, DDP treatment further depleted the intracellular GDF15 protein level in NSCLC cells. However, GDF15 knockdown significantly enhanced the sensitivity of A549/DDP cells to DDP. A possible explanation for this discrepancy relates to the highly context-dependent and secretory nature of GDF15. We postulate that the stress induced by DDP exposure acts as a potent trigger, accelerating the exocytosis of GDF15 out of the tumor cells. Consequently, this secretory event depletes intracellular GDF15 protein abundance, which aligns with the documented accumulation of circulating GDF15 in extracellular fluids.

This dynamic fluctuation of GDF15 expression fundamentally dictates the cellular defense strategy against DDP. Analysis of TCGA data indicates that GDF15 functions as a switch, governing ferroptosis resistance networks. Specifically, high GDF15 expression is associated with suppressors like GPX4, whereas low GDF15 expression correlates with the compensatory transcriptional activation of alternative protective factors, including FANCD2, HSPB1, and SLC7A11. By promoting the post-translational degradation of GDF15, Mor effectively disrupts this adaptive survival mechanism, thereby significantly increasing DDP sensitivity in parental NSCLC cells and mitigating resistance in DDP-resistant cells. Besides its direct action on tumor cell viability, secreted GDF15 is also known to inhibit systemic immune function. Low levels of extracellular GDF15 may facilitate greater T cell infiltration into the tumor tissue (Ozawa et al., 2022). Therefore, abrogating GDF15 through Mor treatment not only overcomes intrinsic chemoresistance via ferroptosis but also provides a rationale for combining DDP with immunotherapies. These findings point to a potential therapeutic strategy of targeting the GDF15-ferroptosis axis for treating DDP-resistant patients, although further studies are warranted to validate these implications in other cancers.

In summary, this work identified a previously unrecognized mechanism of the natural compound Mor in overcoming DDP resistance in NSCLC through directly interacting with GDF15 and promoting its ubiquitin-mediated degradation to facilitate ferroptosis. Our findings suggest a probable pharmacological intervention of Mor-based combination therapy for the treatment of chemoresistant NSCLC.

## Data Availability

The original contributions presented in the study are publicly available. The RNA sequencing data have been deposited in Zenodo and the dataset is publicly accessible via the following DOI/link: https://doi.org/10.5281/zenodo.20148802.
